# Hilbert-domain sub-band feature framework for EEG-based seizure detection

**DOI:** 10.3389/fncom.2026.1843591

**Published:** 2026-06-24

**Authors:** Abdullah Al Shiam, Fahmid Al Farid, Abu Saleh Musa Miah, Md. Humaun Kabir, Hezerul Abdul Karim

**Affiliations:** 1 Department of Computer Science and Engineering, Netrokona University, Netrokona, Bangladesh; 2 Faculty of Computer Science and Informatics, Berlin School of Business and Innovation, Berlin, Germany; 3 Department of Computer Science and Engineering, University of Rajshahi, Rajshahi, Bangladesh; 4 Department of Computer Science and Engineering, Jamalpur Science and Technology University, Jamalpur, Bangladesh; 5 Centre for Image and Vision Computing (CIVC), COE for Artificial Intelligence, Faculty of Artificial Intelligence and Engineering (FAIE), Multimedia University, Cyberjaya, Malaysia

**Keywords:** EEG signal analysis, entropy features, epileptic seizure detection, Hilbert Transform, machine learning, MRMR feature selection

## Abstract

Epilepsy is a chronic neurological disease in which the brain's activity deviates from normal. The classification and analysis of EEG signals is the first step in diagnosing epilepsy. Various machine learning algorithms have been used in the past to classify epileptic EEG recordings. The proposed approach for EEG seizure detection is based on processing signals in the Hilbert domain. In this study, the EEG signal is divided into short time frames. Each frame is decomposed into sub-bands using a Butterworth Bandpass Filter, and the Hilbert Transform is applied to each sub-band of a frame. Next, three categories of features—entropy-based, spike-related, and statistical features—are extracted from each sub-band. A high-dimensional feature vector is produced by concatenating the features obtained from each sub-band. A filter-based technique, Minimum Redundancy Maximum Relevance (mRMR) feature selection method, is used to select a discriminative subset of features. The mRMR method ranks the features based on calculated weights. The proposed framework was evaluated using the publicly available University of Bonn EEG dataset, which contains five EEG classes (A–E), and the CHB-MIT scalp EEG dataset, which consists of long-term pediatric EEG recordings. Experiments conducted on the University of Bonn and Boston Children's Hospital datasets show that the proposed method achieves classification accuracies of 99.27% and 99.04%, surpassing several state-of-the-art methods with performance improvements of up to 1.43%, demonstrating its effectiveness for epileptic seizure detection. Performance evaluation was conducted using repeated 15-fold cross-validation with multiple machine learning classifiers, including Linear SVM, RBF-SVM, Random Forest (RF), Ensemble Tree (ET), and Linear Discriminant Analysis (LDA). Furthermore, Friedman ANOVA and Tukey-Kramer post hoc statistical analyses confirmed the robustness and reliability of the proposed framework.

## 1 Introduction

Epilepsy is a chronic brain illness caused by an abnormal increase in neuronal synchronization. Globally, it has afflicted around 50 million people of all ages (Goldenberg, 2010). The electroencephalogram (EEG) is used to identify various neurological illnesses, including epilepsy. Manually monitoring long-duration EEG readings is monotonous and time-consuming. Thus, an automated epileptic seizure detection system based on modern signal processing and machine learning approaches must be developed to reduce the neurologist's workload and reliably recognize epileptic seizure EEG signals more quickly. An automatic seizure detection system significantly aids in rehabilitation, diagnosis, and long-term epilepsy monitoring (Mormann et al., 2007). EEG signals are inherently nonlinear and non-stationary, particularly during seizure episodes where abrupt amplitude modulation and rapid oscillatory changes occur. Conventional time-domain or purely spectral analysis may fail to fully capture these transient dynamics (Acharya et al., 2013). Therefore, signal representations that provide instantaneous amplitude and phase information are better suited to seizure characterization (Loughlin and Tacer, 1997). We describe our contributions to the proposed work below.

- A Hilbert-domain sub-band feature extraction framework is proposed for epileptic seizure detection, where instantaneous amplitude and phase-related characteristics are extracted from narrowband EEG sub-bands.
- A hybrid feature representation combining entropy-based, spike-related, and statistical features is developed to characterize seizure and non-seizure EEG patterns more effectively.
- An mRMR-based discriminative feature selection strategy is employed to reduce feature redundancy and improve seizure classification performance.
- Extensive evaluation on both the Bonn and CHB-MIT EEG datasets demonstrates that the proposed framework achieves robust and computationally efficient seizure detection performance using conventional machine learning classifiers.
- Statistical significance analysis using Friedman ANOVA and Tukey–Kramer post hoc tests is conducted to validate the robustness of the proposed framework across multiple classifiers.

## 2 Related works

Several automated epileptic seizure detection methods have been published in the literature (Martis et al., 2012; Li et al., 2017). Feature extraction and classification represent two essential stages in an automated epileptic seizure detection system. Various feature extraction techniques based on both time- and frequency-domain analyses have been proposed for detecting epileptic seizures (Martis et al., 2013). Techniques such as the short-time Fourier transform (STFT) have been applied in time-frequency analyses (Srinivasan et al., 2005). Given that EEG signals are non-stationary, time-frequency domain feature extraction is particularly well-suited to studying epileptic seizure signals (Acharya et al., 2013).

An approach based on empirical mode decomposition (EMD) has been developed in Li et al. (2013). Using the EMD approach, the EEG was decomposed into Intrinsic Mode Functions (IMFs), and parameters such as the correlation of variation (CV) and the fluctuation index (FI) were extracted to distinguish seizure from non-seizure. EEG signals were examined using 54-DWT mother wavelets, with feature selection performed via a Genetic Algorithm (GA) and classification carried out using four machine learning classifiers (Mardini et al., 2020). In Mursalin et al. (2017), a method was proposed for identifying epileptic seizures in EEG recordings, utilizing the Improved Correlation-based Feature Selection (ICFS) approach together with a Random Forest (RF) classifier.

The study calculates the weighted multiscale Rényi permutation entropy (WMRPE) from rhythms obtained through the Fourier–Bessel series expansion method (Gupta and Pachori, 2019). The least-square support vector machine (SVM) classifier is then used to classify epileptic EEG signals. A deep learning strategy is used to automatically extract time-, frequency-, and time-frequency-domain features for identifying seizure EEG signals (Solaija et al., 2018). A new method employing Fourier decomposition for epileptic seizure detection in EEG recordings is presented in Mehla et al. (2021). A comprehensive study is presented in Shoeibi et al. (2021), where the authors employed 50 distinct features—including time-domain, statistical, frequency-domain, and nonlinear features—for detecting epileptic seizure EEG signals. In Radman et al. (2020), the combination of time-, frequency-, and time-frequency-domain features with the Dempster–Shafer evidence theory (DSET) for feature selection led to improved seizure classification performance. A one-dimensional deep neural network comprising three convolutional blocks and three fully connected layers is proposed for robust seizure detection (Zhao et al., 2020). This research (Alzamili et al., 2025) introduces an effective feature selection method utilizing Ruzicka similarity for the detection and diagnosis of seizures induced by epilepsy. A fuzzy-based model for epileptic seizure detection was developed in Qureshi et al. (2022), which integrates an innovative feature extraction and selection method with fuzzy classifiers. They extract pattern features using time, frequency, and nonlinear signal analyses.

In this research, the EEG signal is filtered through a Butterworth Bandpass Filter to obtain six frequency sub-bands. Then the Hilbert Transform is applied on each sub-band for feature extraction. The power of the Hilbert Transform lies in its ability to characterize nonlinear and non-stationary signals such as EEG. The Hilbert Transform is used to extract a set of features for the seizure classification scheme. EEG signals representing seizure episodes tend to display a larger number of spike events than signals recorded during non-seizure periods (Ray, 1994). To further describe seizure activity, entropy-based metrics are applied to analyze the complexity of EEG signals. Several studies indicate that using multiple entropy measures in EEG data can significantly improve seizure detection accuracy (Itakura and Tanaka, 2017). Thus, the analysis incorporates four entropy measures in addition to spike-related and statistical characteristics. From each sub-band, ten features are extracted and merged to construct the feature vector. Previous studies have shown that feature fusion improves system performance (Molla et al., 2020b). A combination of spike-detecting, entropy-based, and statistical features enhances the effectiveness of distinguishing EEG signals with seizure events from non-seizure EEG recordings. The increasing number of features degrades the classification performance. The selection of features is essential for increasing the effectiveness of the machine learning-based categorization strategy (Cai et al., 2018). To eliminate redundancy and irrelevance, a smaller subset of features is selected while ensuring that the main objective is maintained or even improved. This approach also helps decrease computational complexity. An appropriate feature selection technique can lead to better classification results (Hoque et al., 2014). A filter-based feature selection method, Minimum Redundancy Maximum Relevance (mRMR), is utilized to extract the most significant features from the high-dimensional feature set for seizure detection. These selected EEG features are then applied to classify seizure and non-seizure signals using various machine learning models, including Linear SVM, RBF-SVM, LDA, Random Forest, and Ensemble Tree classifiers.

## 3 Data description

### 3.1 Bonn EEG dataset

The evaluation of the proposed approach has been conducted using the EEG dataset provided by the Department of Epileptology at the University of Bonn, Germany (Andrzejak et al., 2001). This publicly available dataset contains five classes (labeled A–E) of EEG segments recorded from 100 channels, with each segment lasting 23.6 seconds. For sets A and B, EEG data were recorded from five healthy individuals with a standardized electrode configuration, with eyes open (A) and eyes closed (B). Sets C, D, and E include EEG recordings from five epileptic patients. Sets C and D correspond to patients who had fully recovered from seizure activity following surgical intervention, whereas set E contains EEG signals with active seizure episodes from an epileptogenic region. Noise and artifacts were minimized using a 0.53–40 Hz bandpass filter, which also attenuates power-line noise. The recordings were obtained via a 128-channel amplifier at a sampling frequency of 173.61 Hz. In our detection system, we focus on differentiating between the healthy subjects and ictal (seizure) subjects, interictal (non-seizure) subjects, and ictal (seizure) subjects.

### 3.2 CHB-MIT dataset

This research was conducted using the CHB-MIT database from Children's Hospital Boston (Ali et al., 2024). EEG recordings from 24 pediatric patients with intractable seizures are included in this dataset, totaling 916 h of data and 23 sessions from 22 patients aged 1.5 to 22 years. From the CHB-MIT database, 664 EEG files encompassing 198 seizure events were labeled as seizure or non-seizure recordings. Signals were captured at a sampling rate of 256 Hz and 16-bit resolution, with each file spanning either 1 or 4 hours. Among these, 129 files contained one or more seizure occurrences. The recordings employed the International 10–20 system for electrode placement. Given the large dataset, EEG signals were segmented using temporal windows for further analysis.

## 4 Methodology

**Figure 1:**
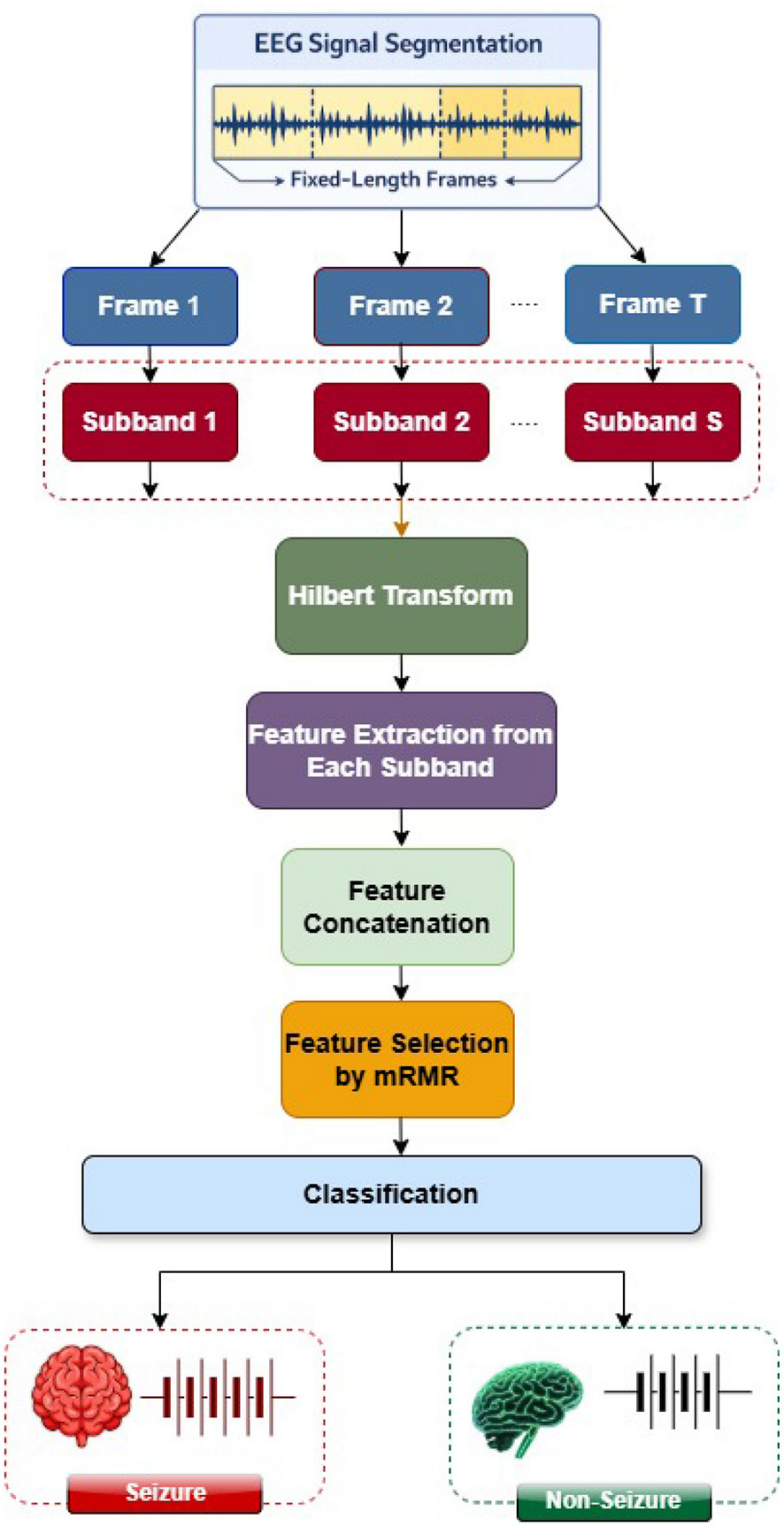
Block diagram of the proposed method.

In this research, a Hilbert-domain-based sub-band feature extraction technique is proposed together with a feature selection process to differentiate epileptic from non-epileptic EEG signals using machine learning classifiers. The block diagram of the automatic epileptic seizure detection system is shown in Figure 1. It consists of the following steps:

1. Each EEG signal segment is separated into frames of equal length.
2. Each frame is separated into several sub-bands for further analysis (1–6 Hz, 6–11 Hz, 11–22 Hz, 22–43 Hz, 43–86, 1–86 Hz) using a Butterworth Bandpass Filter.
3. Hilbert Transform is applied on each sub-band of a frame.
4. Each frame's sub-bands are analyzed to extract four entropy-based features—Shannon, Rényi, log energy, and Tsallis—alongside three spike-related and three statistical features.
5. To derive the feature vector, all the extracted features are combined.
6. To select the features that best discriminate between classes, the minimum redundancy maximum relevance (mRMR) feature selection technique is applied.
7. Using the selected features, various machine learning classifiers are trained to distinguish EEG frames corresponding to ictal activity from those representing interictal or healthy states.

The signal is divided into equal halves for framing each EEG segment. The frame is set to 11.8 seconds for the Bonn dataset and 8 seconds for the CHB-MIT dataset, without overlap. These lengths were chosen based on prior studies and empirical evaluation to ensure sufficient seizure-related content within each segment while maintaining computational efficiency. Non-overlapping windows were used to simplify feature extraction and avoid redundant data. However, we acknowledge that overlapping windows could improve temporal resolution and reduce potential information loss at segment boundaries. Future work could incorporate overlapping segmentation to capture finer temporal dynamics. The selection of window length, overlap ratio, and filter configuration can significantly influence EEG seizure detection performance. Shorter window lengths may provide faster seizure localization and better temporal resolution; however, they may contain insufficient discriminative information and increase signal variability. Conversely, larger windows can capture more stable seizure characteristics but may reduce temporal sensitivity and increase computational complexity. Similarly, overlapping windows may improve continuity between adjacent EEG segments and reduce boundary information loss, although they also increase computational cost and data redundancy. The proposed framework is divided into two parts. The first part includes a preprocessing step for extracting relevant features, followed by classification. In the first part, sub-band signals are obtained using the Butterworth Bandpass Filter method for EEG signals. The EEG sub-band decomposition (1–6 Hz, 6–11 Hz, 11–22 Hz, 22–43 Hz, 43–86 Hz, and 1–86 Hz) was applied to keep the preprocessing consistent and to preserve a wide range of EEG frequencies. For the University of Bonn EEG dataset, the upper cutoff is close to the Nyquist limit (86.8 Hz), but most of the important information is below 40–60 Hz, so any effects from the high-frequency boundary are minimal. For the CHB-MIT EEG dataset, the chosen range is comfortably within the Nyquist limit, ensuring a stable signal. The extracted sub-bands do not exactly match traditional EEG frequency ranges, but they were chosen based on previous studies for effective seizure feature detection. Each sub-band is created using a third-order Butterworth Bandpass Filter, which has a flat passband and a moderate roll-off. The filter uses a 2 Hz transition bandwidth, 0.5 dB passband ripple, and 40 dB stopband attenuation. Filter configurations, including different filter orders, transition bandwidths, and frequency ranges, may affect the preservation of seizure-related frequency components and noise suppression capability. Therefore, future work will investigate adaptive windowing strategies, optimized overlap ratios, and alternative filter designs to further improve the robustness and generalization capability of the proposed framework. The sub-bands of each frame are transformed using the Hilbert Transform, and features are subsequently obtained from the Hilbert domain. In the second step, feature selection is performed using the filter-based mRMR approach to identify a potential subset of features. The proposed method's performance is then evaluated using machine learning classifiers to distinguish seizure EEG signals.

### 4.1 Sub-band decomposition

**Figure 2:**
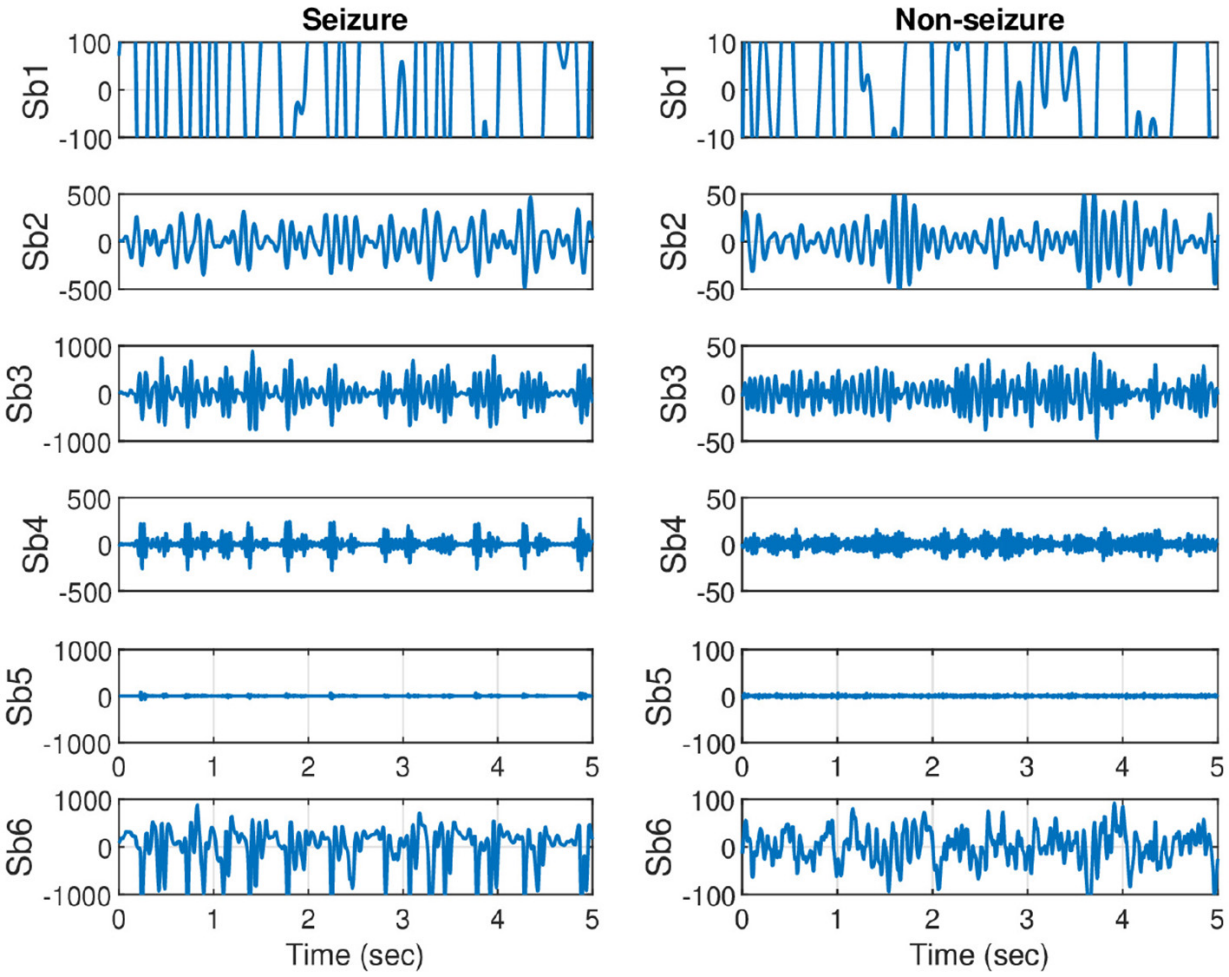
Sub-band signals (Sb1–Sb6) of a 5s EEG segment, obtained using the Butterworth Bandpass Filter from set A (non-seizure) and set E (seizure) of the Bonn EEG dataset.

**Figure 3:**
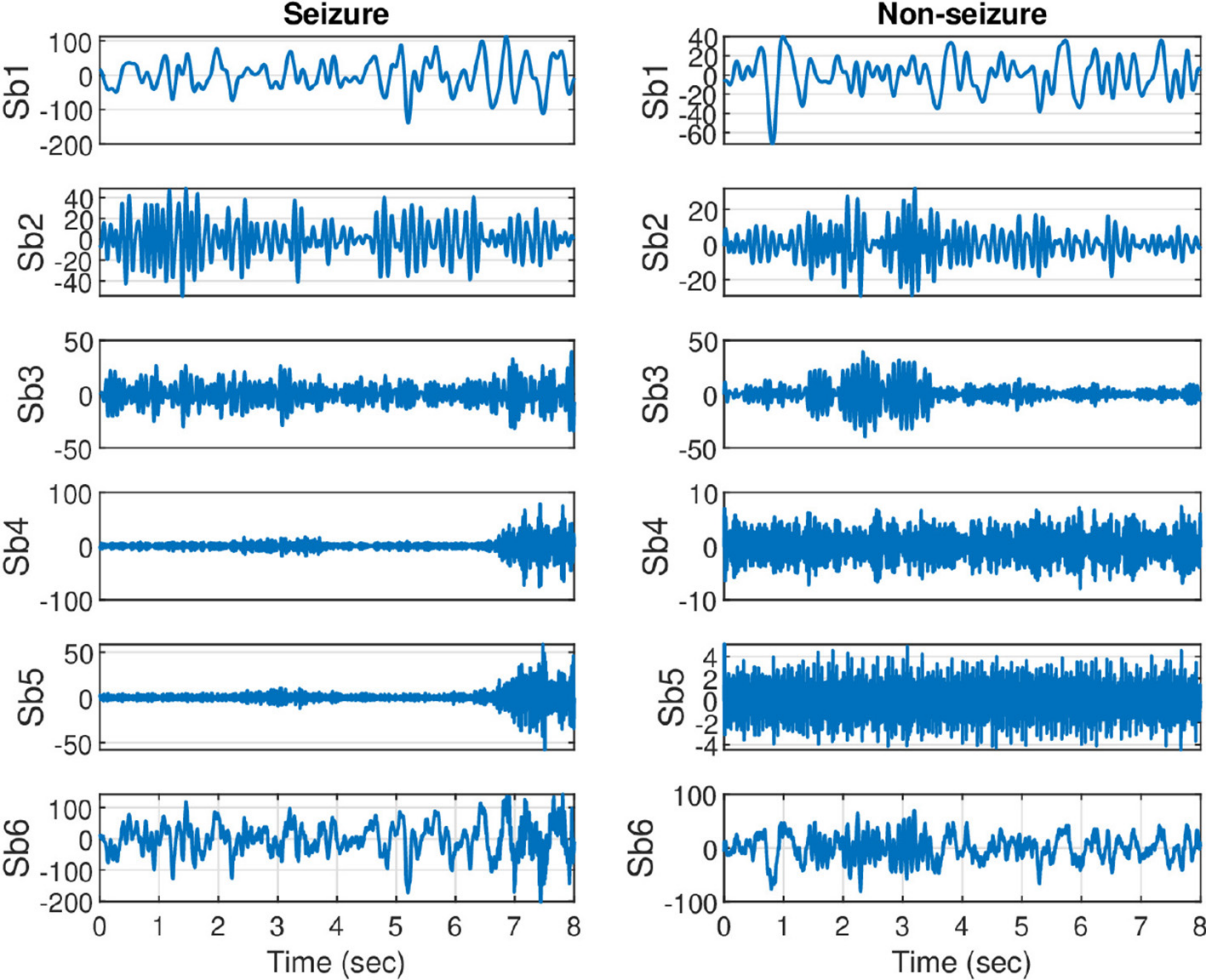
Sub-band signals (Sb1–Sb6) of an 8 s EEG segment obtained using the Butterworth Bandpass Filter from Chb01 subject of the CHB-MIT EEG dataset.

In this study, each EEG segment was processed using a third-order Butterworth Bandpass Filter to obtain the narrowband component shown in Figures 2, 3. To balance computational efficiency and frequency selectivity, a third-order Butterworth filter was selected. The Butterworth filter maintains the morphological features of EEG signals without causing significant amplitude distortion, thanks to its maximum flat passband response. Although higher-order filters can offer more precise frequency separation, they may also introduce phase distortion and increased computational complexity. However, lower-order filters might not be enough to separate the EEG sub-bands. Therefore, the third-order Butterworth filter is appropriate for the suggested framework's stable and effective EEG sub-band decomposition. Several studies have suggested sub-band decomposition to improve detection outcomes. Multiple filters with different passbands were utilized in those investigations (Molla et al., 2020b; Akter et al., 2020). Features extracted from narrow frequency bands provide key insights for identifying seizure events (Mursalin et al., 2017; Wang et al., 2011). The Butterworth Bandpass Filter effectively analyzes most physiological signals exhibiting nonstationary patterns (Daud and Sudirman, 2015). In our study, six sub-band extracts at sampling frequencies of 173.61 Hz and 256 Hz were used for the Bonn and CHB-MIT datasets.

The Hilbert Transform provides meaningful instantaneous frequency estimation primarily when applied to narrowband or mono-component signals. Applying it directly to broadband EEG may violate this assumption, leading to ambiguous interpretations. Therefore, sub-band decomposition is performed before Hilbert Transformation to ensure that each component satisfies the narrowband condition and preserves physiologically meaningful oscillatory behavior.

### 4.2 Hilbert Transform over decomposition

The Hilbert Transform was performed on the components after bandpass filtering. The Hilbert Transform converts the original time-series signal *X*(*t*) into another time-series signal *S*(*t*). Hilbert Transform is the convolution between Hilbert Transformer (1/±π*t*) and the signal *X*(*t*). The Hilbert Transform is applied to *X*(*t*) to produce *X*_*H*_(*t*), as shown in Equation 1.

The analytic signal corresponding to a real-valued EEG sub-band signal *x*(*t*) can be represented as:


$$\begin{array}{l} z\left(t\right)=x\left(t\right)+jH\left\{x\left(t\right)\right\} \end{array}$$ (1)

where $j=\sqrt{-1}$, *x*(*t*) is the real-valued EEG signal, and H{x(t)} denotes the Hilbert Transform of *x*(*t*). The Hilbert Transform is defined as:


$$\begin{array}{l} H\left\{x\left(t\right)\right\}=\frac{1}{\pi }\text{P}.\text{V}.\int_{-\infty }^{\infty }\frac{x\left(\tau \right)}{t-\tau }d\tau \end{array}$$ (2)

where P.V. represents the Cauchy principal value. The analytic signal can also be expressed in polar form as:


$$\begin{array}{l} z\left(t\right)=A\left(t\right)e^{j\phi \left(t\right)} \end{array}$$ (3)

where *A*(*t*) = |*z*(*t*)| represents the instantaneous amplitude and ϕ(*t*) = arg(*z*(*t*)) represents the instantaneous phase.

Seizure EEG signals are characterized by sudden amplitude bursts and phase irregularities. The analytic signal representation obtained via the Hilbert Transform provides instantaneous amplitude and phase information, thereby enhancing the detection of such transient events. These instantaneous measures are more sensitive to spike morphology compared to conventional statistical or spectral descriptors.

The analytical form denotes a π/2 phase shift or phase difference between the positive and negative frequencies. The imaginary component representing negative frequencies is omitted due to Hermitian symmetry, and only the real component corresponding to positive frequencies is used. The derivative of the instantaneous phase, or instantaneous frequency, is defined as ω(*t*) = φ(*t*)′, where the prime represents the differentiation (Loughlin and Tacer, 1997). Here ω(*t*) denotes the instantaneous frequency of the EEG signal. The extracted Hilbert-domain representation is subsequently used to extract entropy-based, spike-related, and statistical features for seizure detection.

### 4.3 Feature extraction

The feature extraction technique reduces the amount of data that must be processed while retaining relevant information. This study incorporates three types of features, and their integration is employed to represent EEG signals in relation to seizure events. To obtain features from each segment, the *n*-th sub-band is represented by *X*, which can be written as *X* = *x*_1_, *x*_2_, ..., *x*_*l*_, where *l* is the length of *X*. The features are described in detail below.

#### 4.3.1 Shannon Entropy

Shannon entropy can express the amount of information provided by an event when it occurs. The entropy of a signal *X* is normally defined as:


$$\begin{array}{l} S_{En}=-\overset{l}{\underset{i=1}{\sum }}p\left(x_{i}\right)\log\left(p\left(x_{i}\right)\right) \end{array}$$ (4)

For each EEG epoch, the signal is first squared to obtain its instantaneous energy representation. A normalized energy distribution is then computed as:


$$\begin{array}{l} P_{i}=\frac{x_{i}^{2}}{\sum_{j=1}^{N}x_{j}^{2}} \end{array}$$ (5)

where *P*_*i*_ is the probability associated with the *i*th sample. N is the number of data points. $x_{i}^{2}$ is the energy of the current sample.

#### 4.3.2 Renyi's Entropy

Rényi's entropy has frequently been used in research to distinguish seizure activity (Mammone et al., 2015; Hadiyoso et al., 2019; Yol et al., 2018). It is defined as:


$$\begin{array}{l} R_{En}\left(\gamma \right)=\frac{1}{1-\gamma }\log\overset{l}{\underset{i=1}{\sum }}{\left(p_{i}\right)}^{\gamma },\gamma >0,\gamma \neq 1 \end{array}$$ (6)

where *p*_*i*_ represents the normalized power spectral density, here we use γ = 2.

#### 4.3.3 Tsallis Entropy

Tsallis Entropy is a measure of uncertainty about an event (Tsallis, 1988). If *p*_*i*_ is the set of probabilities and γ any real number, it can be defined as:


$$\begin{array}{l} TS_{En}\left(\gamma \right)=1-\frac{\sum_{i=1}^{l}{\left(p_{i}\right)}^{\gamma }}{\gamma -1} \end{array}$$ (7)

#### 4.3.4 Log Energy Entropy

Log Energy Entropy is used to characterize the non-linear dynamics of EEG signals (Aydın et al., 2009). The log-energy entropy corresponding to the signal X is given by:


$$\begin{array}{l} \log_{En}=\overset{l}{\underset{i=1}{\sum }}{\left(\log\left(p\left(x_{i}\right)\right)\right)}^{2} \end{array}$$ (8)

#### 4.3.5 Fluctuation Index

The Fluctuation Index is a mathematical metric that determines how much the amplitude of a signal changes over time (Molla et al., 2020a; Li et al., 2013). *F*_*I*_ is defined as:


$$\begin{array}{l} F_{I}=\frac{1}{l-1}\overset{l-1}{\underset{i=1}{\sum }}|x_{i+1}-x_{i}| \end{array}$$ (9)

#### 4.3.6 Coefficient of variation

In various epilepsy studies, the coefficient of variation (*C*_*V*_) was used to characterize signal characteristics (Akter et al., 2020).


$$\begin{array}{l} C_{V}sb=\frac{\sigma }{\bar{x}}\times 100\% \end{array}$$ (10)

where σ is the standard deviation and $\bar{x}$ is the mean of the data.

#### 4.3.7 Second order difference plot (SODP)

SODP, along with the computation of the ellipse area, is utilized in this study. SODP can generate informative diagnostic features that help classify EEG signals as seizure or non-seizure. It is often used to detect spike events in the signal (Molla et al., 2020a; Hassan et al., 2019). For signal *X*, the SODP, which involves plotting *Y*_1_ against *Y*_2_, can be defined as:


$$\begin{array}{l} Y_{1}=x_{i+1}-x_{i} \end{array}$$ (11)

$$\begin{array}{l} Y_{2}=x_{i+2}-x_{i+1} \end{array}$$ (12)

Using the parameters *a* and *b*, which represent the major and minor radii of the 95% confidence ellipse, the area of the ellipse can be calculated as follows:


$$\begin{array}{l} A_{e}=\pi ab \end{array}$$ (13)

The detailed calculation of the 95% confidence ellipse area of SODP can be found in Molla et al. (2020a).

#### 4.3.8 Kurtosis

It measures the spikiness of a signal (Xiang et al., 2021). The formula is defined as follows:


$$\begin{array}{l} K_{X}=\frac{\frac{1}{l}\sum_{i=1}^{l}{\left(x_{i}-\mu \right)}^{4}}{{\left(\frac{1}{l}\sum_{i=1}^{l}{\left(x_{i}-\mu \right)}^{2}\right)}^{2}}-3 \end{array}$$ (14)

#### 4.3.9 Skewness

In various studies, skewness was used to quantify the asymmetry of signals (Xiang et al., 2021). This can be defined as:


$$\begin{array}{l} S_{Q}=\frac{\frac{1}{N}\sum_{i=1}^{N}{\left(x_{i}-\bar{x}\right)}^{3}}{{\left(\frac{1}{N}\sum_{i=1}^{N}{\left(x_{i}-\bar{x}\right)}^{2}\right)}^{3/2}} \end{array}$$ (15)

where *x*_*i*_ represents each data point. $\bar{x}$ is the mean, and N is the total number of data points.

#### 4.3.10 Hjorth activity

This parameter represents the signal power and variance of a time function and can be formulated as follows. It can be represented by the following equations:

- **Activity** (Variance of amplitude):

$$\begin{array}{l} \text{Activity}=\text{var}\left(x\right) \end{array}$$ (16)

- **Mobility** (Mean frequency):

$$\begin{array}{l} \text{Mobility}=\sqrt{\frac{\text{var}\left(ẋ\right)}{\text{var}\left(x\right)}} \end{array}$$ (17)

- **Complexity** (Signal bandwidth):

$$\begin{array}{l} \text{Complexity}=\frac{\text{Mobility}\left(ẋ\right)}{\text{Mobility}\left(x\right)}=\frac{\sqrt{\text{var}\left(ẍ\right)/\text{var}\left(ẋ\right)}}{\sqrt{\text{var}\left(ẋ\right)/\text{var}\left(x\right)}} \end{array}$$ (18)

where ẋ is the first derivative and ẍ is the second derivative of the signal.

### 4.4 Feature combination

EEG segments are filtered through a Butterworth Bandpass Filter to obtain six sub-bands. We computed features for each sub-band using the above feature-extraction methods. Therefore, we have (6 sub-bands * 10 features) 60 features that are combined to derive a feature vector *F*_*C*_ applied during feature selection, contributing to the detection of seizures.

### 4.5 Discriminative feature selection

Feature selection involves selecting the most relevant features for a given task by ranking them according to their contribution to the classification model. Most feature selection schemes consider only the relationship between features and classification categories, ignoring the mutual interaction of features. Instead, the mRMR feature selection algorithm used in this study takes into account not only the amount of information provided by these features for categorical attributes but also the impact of feature interaction on classification (Peng et al., 2005; Kabir et al., 2023). Let *F*_*C*_ be a set of features, the minimum redundancy condition is defined as:


$$\begin{array}{l} min_{RD}=\frac{1}{|F_{C}{|}^{2}}\underset{v_{i},v_{j}\in F_{C}}{\sum }I\left(v_{i},v_{j}\right) \end{array}$$ (19)

where, *I*(*v*_*i*_, *v*_*j*_) represents the mutual information between features *v*_*i*_ and *v*_*j*_ and *p* represents the probability function. The maximum relevance condition is defined as:


$$\begin{array}{l} max_{RE}=\frac{1}{\left|F_{C}\right|}\underset{v_{i}\in F_{C}}{\sum }I\left(v_{i},h\right) \end{array}$$ (20)

where *I*(*v*_*i*_, *h*) is the mutual information between feature *v*_*i*_ and the class label. Continuous features were discretized into 10 equal-width bins, and mutual information between each feature and the target class was calculated based on these probability distributions. The Mutual Information Difference (MID) criterion was used to iteratively select features, maximizing relevance to the target while minimizing redundancy with previously selected features. The number of selected features was optimized for classification performance.

Before classification, extracted features were normalized using z-score normalization to reduce scale-related bias among heterogeneous feature groups. During each cross-validation iteration, the normalization parameters (mean and standard deviation) were computed from the training data and then applied to the corresponding test data to avoid information leakage. After normalization, mRMR feature selection was performed on the training set to select the most discriminative features.

### 4.6 Classifiers

In previous research, several machine learning classifiers were used to distinguish normal EEG signals from epileptic seizure signals (Martis et al., 2012), including an ANN (Molla et al., 2020a). Here we have experimented with five different classifiers: Linear SVM, RBF-SVM, Linear Discriminant Analysis, Random Forest, and Ensemble Tree.

#### 4.6.1 Support vector machine

The Support Vector Machine (SVM) is a supervised learning technique that identifies an ideal hyperplane to separate classes with the largest possible margin. It effectively handles high-dimensional and nonlinearly separable data by employing kernel functions. Linear SVM and non-linear RBF-SVM were evaluated in this study. As the main classifier, the Linear SVM was chosen for its strong generalization, computational efficiency, and suitability for high-dimensional EEG feature spaces. To further investigate the effect of non-linear decision boundaries, an RBF-SVM classifier was also implemented for comparison. During training, the hyperparameters of the RBF-SVM, including BoxConstraint (C) and KernelScale, were optimized using 5-fold cross-validation. The last set of optimized parameters was automatically selected based on classification performance.

#### 4.6.2 Linear Discriminant Analysis

Linear Discriminant Analysis (LDA) is a statistical classification technique that projects data into a lower-dimensional space to enhance class separation. It presumes classes that are normally distributed with identical covariance matrices. The LDA classifier was implemented using MATLAB's built-in discriminant analysis function with default settings and equal covariance assumptions across classes.

#### 4.6.3 Random forest

Breiman (Breiman, 2001) was the first to introduce the Random Forest classifier, which is a combination of numerous decision trees. In a Random Forest model, every tree is constructed independently and contributes to the final decision. Each time, a random sample is taken from the input sample and passed to the trees. Among all the trees, the most popular vote is considered the classification output. Unlike an Ensemble Tree, not all the training data will be used. The Random Forest classifier was implemented using 20 decision trees with bootstrap aggregation and out-of-bag prediction enabled during training.

#### 4.6.4 Ensemble tree

Ensemble tree is a new version of the bagging method and is also called a randomized tree classifier. In ET, the entire sample is used to build each tree. The decision trees are derived using this method by building base classifiers on subsets of samples from the original data, with replacement. The Ensemble Tree classifier was implemented using bagging with decision trees, where the maximum number of splits for each tree was set to 20.

The RBF-SVM classifier was only optimized for its hyperparameters, as nonlinear kernels are more sensitive to parameter selection. For training, an internal 5-fold cross-validation strategy was used to automatically tune the BoxConstraint and KernelScale parameters. Linear SVM, LDA, RF, and ET employed fixed classifier configurations due to their low sensitivity to hyperparameter variations and computational efficiency.

## 5 Experimental results

We have used MATLAB to implement the proposed framework. The programming has been carried out on a system with an Intel Core i5 processor and 16 GB of RAM, running Windows 11. In this study, experiments are conducted using well-known, publicly available EEG datasets to classify seizure vs. non-seizure signals, as defined in Section 3. In this work, six cases are generated from the Bonn dataset for signal classification, as shown in Table 1. The dataset was segmented into fixed frames. A Butterworth Bandpass Filter is used to decompose the signal into narrow, non-overlapping sub-band signals of various bandwidths. Five sub-band signals are obtained from each frame. As an additional sub-signal, the fullband signal is also kept. Thus, each frame contains a set of six sub-signals. Each of the six sub-signals representing a frame is used to extract the features. The six sets of features thus obtained are combined to form the feature vector. The subset of features is selected using the mRMR feature selection method. Multiple learning classifiers are used to classify the signal as epileptic vs. non-epileptic. The performance of the classifier is quantified by accuracy (ACC), sensitivity (SEN), specificity (SPEC), precision (PREC), F1-Score (F1), and false positive rate (FPR) in terms of percentage (%) defined as: *ACC* = 100 × (*TP*+*TN*)/(*TP*+*TN*+*FP*+*FN*), *SEN* = 100 × *TP*/(*TP*+*FN*), *SPEC* = 100 × *TN*/(*TN*+*FP*) respectively, where *TP* represents correctly detected positive, *TN* represents correctly detected negative, *FP* represents falsely detected positive, *FN* represents falsely detected negative. To ensure reliability and robustness of the proposed method, k-fold (*k* = 15) fold cross-validation is used. Where the feature vector is divided into fifteen subsets, and one subset is used for testing, and the remaining subsets are used for training purposes. For example, there are 400 frames (A–E), of which 373 (93.25%) frames are used in training, and 27 (6.75%) are used for validation. We repeated this process 100 times, and the average accuracy across those runs is used for comparison.

**Table 1:** Different cases derived for the classification of the Bonn dataset.

Class	Case					
	1	2	3	4	5	6
Non-seizure	A	B	C	D	AB	CD
Seizure	E	E	E	E	E	E

### 5.1 Performance evaluation of Bonn EEG dataset

The proposed method's classification performance is shown in Table 2 using five different classifiers, ET, RF, Linear SVM, RBF-SVM, and LDA, across six binary classification cases in terms of ACC, SEN, SPEC, PREC, F1, and FPR.

**Table 2:** Classification performances of the proposed method using different machine learning classifiers on the Bonn dataset in terms of ACC (%), SEN (%), SPEC (%), PREC (%), F1-score (%), and FPR (%).

Classifier	Metric	Case 1 (A–E)	Case 2 (B–E)	Case 3 (C–E)	Case 4 (D–E)	Case 5 (AB–E)	Case 6 (CD–E)	Average ± STD
ET	PREC	100.00	99.53	98.73	99.37	99.53	98.58	99.29 ± 0.49
	F1	100.00	99.50	98.72	98.45	99.50	97.74	98.99 ± 0.76
	FPR	0.00	0.50	1.36	0.68	0.25	0.76	0.59 ± 0.43
	SEN	98.98	98.23	98.74	97.13	98.45	96.12	97.94 ± 1
	SPEC	99.00	98.89	98.63	99.01	99.34	97.86	98.79 ± 0.46
	ACC	99.06	97.56	98.68	97.67	98.34	98.07	98.23 ± 0.53
RF	PREC	100.00	99.57	98.81	99.12	99.56	98.35	99.24 ± 0.54
	F1	99.98	99.50	98.74	98.28	99.50	97.48	98.91 ± 0.85
	FPR	0.00	0.46	1.27	0.95	0.23	0.88	0.63 ± 0.44
	SEN	99.94	99.47	98.77	97.66	99.38	96.79	98.67 ± 1.1
	SPEC	100.00	99.55	98.70	99.08	99.76	98.97	99.34 ± 0.46
	ACC	99.97	99.51	98.73	98.37	99.63	98.24	99.08 ± 0.66
RBF-SVM	PREC	99.84	99.59	99.07	98.67	99.57	98.60	99.22 ± 0.47
	F1	99.77	99.50	98.82	98.24	99.37	97.96	98.94 ± 0.66
	FPR	0.20	0.45	0.99	1.21	0.23	0.75	0.64 ± 0.38
	SEN	99.73	99.46	98.66	97.81	99.22	97.47	98.73 ± 0.84
	SPEC	99.80	99.55	99.01	98.79	99.77	99.25	99.36 ± 0.38
	ACC	99.76	99.50	98.83	98.30	99.58	98.66	99.11 ± 0.54
Linear SVM	PREC	99.95	99.57	99.06	99.04	99.72	98.82	99.36 ± 0.41
	F1	99.92	99.55	98.79	98.44	99.47	97.91	99.01 ± 0.7
	FPR	0.05	0.46	1.00	1.04	0.15	0.63	0.56 ± 0.38
	SEN	100.00	99.65	98.71	97.94	99.34	97.19	98.81 ± 0.98
	SPEC	100.00	99.86	99.03	99.29	99.88	99.40	99.58 ± 0.36
	ACC	100.00	99.75	98.87	98.61	99.70	98.67	99.27 ± 0.56
LDA	PREC	100.00	100.00	99.77	99.75	100.00	98.52	99.67 ± 0.53
	F1	100.00	99.05	99.39	98.10	98.58	95.70	98.47 ± 1.38
	FPR	0.00	0.01	0.24	0.25	0.00	0.77	0.21 ± 0.27
	SEN	99.99	98.21	99.06	96.63	97.25	93.23	97.40 ± 2.17
	SPEC	100.00	100.00	99.78	99.73	100.00	99.24	99.80 ± 0.27
	ACC	100.00	99.10	99.42	98.17	99.08	97.24	98.84 ± 0.9

Overall, all classifiers achieved very high performance, with average accuracies exceeding 98%, demonstrating the effectiveness of the selected feature set for seizure detection. Among the evaluated models, Linear SVM achieved the best overall performance, with the highest average accuracy of 99.27%, average sensitivity of 98.81%, and average specificity of 99.58%. Furthermore, Linear SVM achieved superior precision and F1-score values while maintaining a very low false positive rate, indicating its strong capability in correctly detecting seizure EEG signals with minimal false alarms. Notably, Linear SVM achieved 100% accuracy in Case 1 (A vs. E), indicating excellent separability between normal and seizure EEG signals. The RBF-SVM classifier also exhibited a very competitive performance in all cases. The nonlinear kernel effectively handled complex decision boundaries in the EEG feature space and improved classification performance in several difficult cases. Despite its good performance, the average results of RBF-SVM were slightly lower than those of Linear SVM in terms of overall stability and computational simplicity. Random Forest (RF) also demonstrated better performance, with high precision and F1-score values, and achieved an average accuracy of 99.08%, closely following Linear SVM. ET achieved competitive results, with an average accuracy of 98.23%. Although LDA achieved perfect accuracy in Case 1, its overall average performance (98.84%) was slightly lower compared to Linear SVM and RF, particularly in more challenging cases such as Case 6 (CD vs. E), where sensitivity decreased. Across all classifiers, specificity values were consistently high (mostly above 99%), indicating strong ability to correctly identify non-seizure signals. However, sensitivity showed relatively larger variation, especially in combined cases (AB vs. E and CD vs. E), suggesting that these cases are comparatively more challenging. In summary, the results confirm that while ensemble methods (ET and RF) provide robust performance, Linear SVM offers the most consistent and superior classification results for the proposed approach.

#### 5.1.1 Statistical significance analysis

Using the classification accuracies acquired across several runs, we used Friedman's non-parametric ANOVA test and the Tukey–Kramer post hoc test to identify the statistically significant differences among the classifiers of observed performance. We used the Friedman test because it does not assume normality and is appropriate for comparing multiple classifiers over repeated experiments. The Friedman ANOVA test on the Bonn EEG dataset yielded *p*-values < 0.05 for all six classification cases (A–E, B–E, C–E, D–E, AB–E, and CD–E), indicating that the evaluated classifiers differed significantly. A later Tukey-Kramer post hoc analysis is shown in Table 3, which shows that Linear SVM and RBF-SVM generally outperformed LDA with statistically significant differences in most cases. However, in several cases, the differences in performance among Linear SVM, Ensemble Tree, and Random Forest were relatively small, indicating that these classifiers performed similarly. In difficult classification cases, such as CD–E, Linear SVM differed significantly from Random Forest, and LDA differed significantly from the other classifiers, which supports the increased robustness of the SVM-based classifiers.

**Table 3:** Friedman ANOVA and Tukey-Kramer post hoc statistical analysis for different classifiers on the Bonn EEG dataset.

Case	Friedman *p*-value	Significant difference	Best classifier	Comparable classifiers
A vs. E	< 0.001	Yes	Linear SVM	ET, RF
B vs. E	< 0.001	Yes	Linear SVM	RBF-SVM, ET, RF
C vs. E	< 0.001	Yes	LDA	Linear SVM, RBF-SVM
D vs. E	< 0.001	Yes	Ensemble Tree	Linear SVM, RBF-SVM
AB vs. E	< 0.001	Yes	RF/ET	Linear SVM
CD vs. E	< 0.001	Yes	RBF-SVM	Linear SVM, ET

Classifiers listed under Comparable Classifiers did not show statistically significant performance differences according to the Tukey–Kramer post-hoc analysis (*p*>0.05).

#### 5.1.2 Quantitative performance comparison

To further investigate the effectiveness of the proposed Hilbert-domain feature framework, three deep learning models were also implemented, including Convolutional Neural Network (CNN), Long Short-Term Memory (LSTM), and Transformer. The same EEG segments and evaluation protocol used for the machine learning classifiers were applied to these models. Table 4 presents the classification accuracy (%) of the Linear SVM, CNN, LSTM, and Transformer models across different seizure detection cases using the Bonn EEG dataset. For the A vs. E case, Linear SVM achieved the highest accuracy of 100%, followed closely by the Transformer (99.50%). The CNN obtained 93.50%, while LSTM showed comparatively lower performance (58.50%). In the B vs. E case, Linear SVM again achieved superior performance (99.75%), while the Transformer and CNN obtained 98.00% and 96.00%, respectively. LSTM achieved 72.50%. For the C vs. E and D vs. E classification tasks, Linear SVM consistently achieved high accuracies of 98.87% and 98.61%, respectively. The Transformer achieved 83.50% and 90.50%, while CNN maintained stable performance around 93%–94%. LSTM showed the lowest performance in these cases. In grouped cases (AB vs. E and CD vs. E), Linear SVM and Transformer demonstrated strong performance, achieving 99.70% and 99.33% for AB vs. E and 98.67% and 96.00% for CD vs. E, respectively. Overall, Linear SVM achieved the highest average accuracy of 99.27%, followed by CNN (94.61%) and Transformer (94.47%), while LSTM achieved the lowest average accuracy (61.86%).

**Table 4:** Classification accuracy (%) comparison of Linear SVM and deep learning models on the Bonn EEG dataset for different seizure detection cases.

Case	Linear SVM	CNN	LSTM	Transformer
A vs. E	100.00	93.50	58.50	99.50
B vs. E	99.75	96.00	72.50	98.00
C vs. E	98.87	93.00	50.00	83.50
D vs. E	98.61	94.50	50.50	90.50
AB vs. E	99.70	97.33	73.00	99.33
CD vs. E	98.67	93.33	66.67	96.00
**Average** **±** **STD**	**99.27** **±** **0.56**	**94.61** **±** **1.57**	**61.86** **±** **9.50**	**94.47** **±** **5.77**

Linear SVM and deep learning models on the Bonn EEG dataset for different seizure detection cases. Bold values indicate the best performance.

#### 5.1.3 ROC and AUC analysis

**Figure 4:**
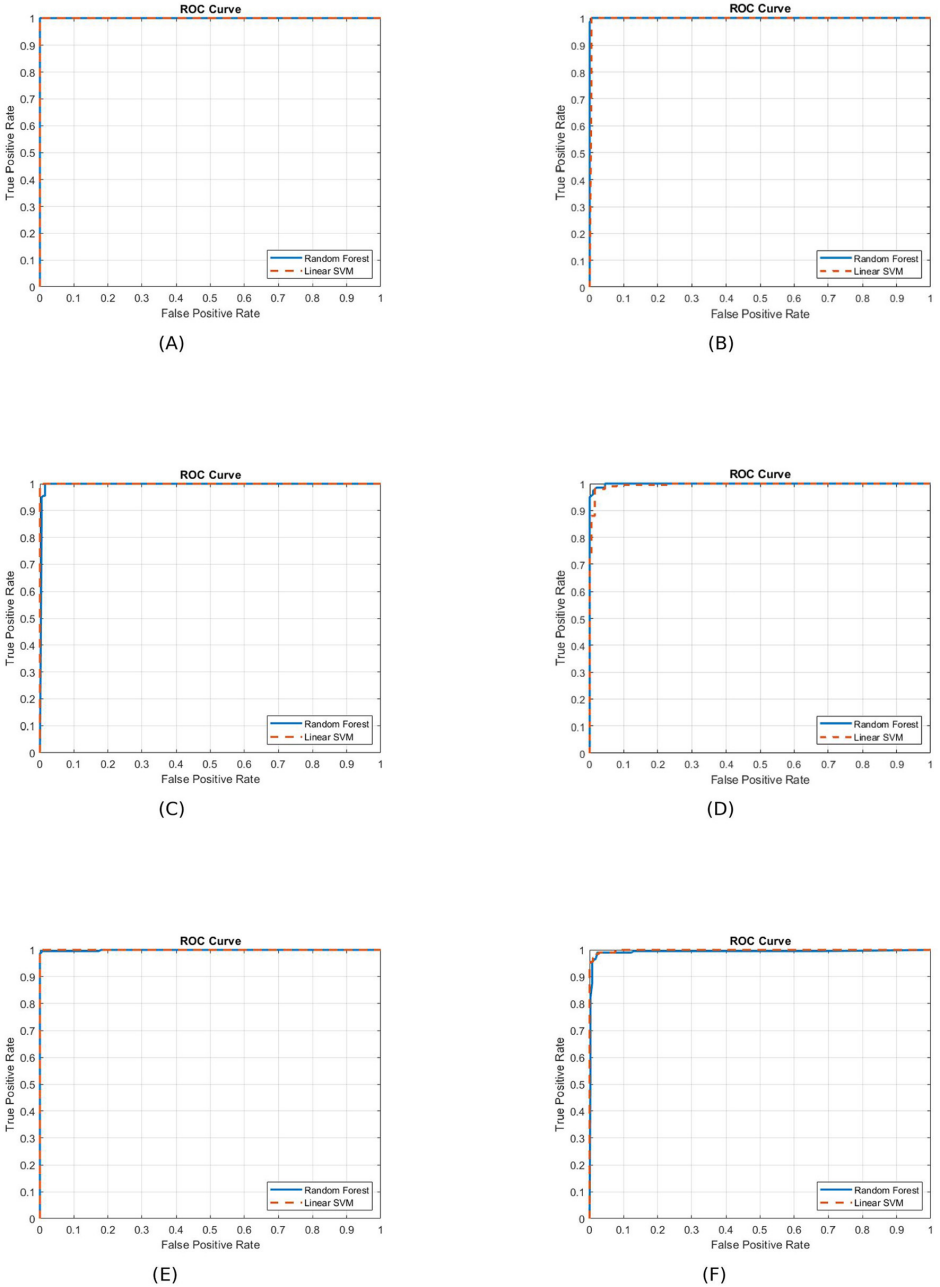
ROC curves for different seizure detection cases on the Bonn EEG dataset. **(A)** A vs. E. **(B)** B vs. E. **(C)** C vs. E. **(D)** D vs. E. **(E)** AB vs. E. **(F)** CD vs. E.

The Receiver Operating Characteristic (ROC) curves for all the classification cases are presented in Figure 4. As illustrated, the ROC curves for the Linear SVM and Random Forest models closely approach the top-left corner of the ROC space, indicating excellent discriminative capability. For the A vs. E case, the ROC curve demonstrates near-perfect classification performance, with the curve almost coinciding with the ideal classification boundary. A similar thing is observed for the B vs. E and AB vs. E cases, where the true positive rate remains close to 1 across nearly all false positive rate values. In the C vs. E, D vs. E, and CD vs. E cases, the ROC curve also exhibits strong separability. However, minor deviations from the perfect boundary can be observed compared to other cases. Overall, the ROC analysis confirms the high classification performance shown in Figure 4, particularly for the Linear SVM and Random Forest models.

Table 5 presents the Area Under the Curve (AUC) values for Linear SVM and RF across the six classification cases. The AUC metric evaluates the classifier's ability to discriminate between seizure and non-seizure EEG signals independent of the decision threshold. Both classifiers achieved exceptionally high AUC values (greater than 99%) in all cases, indicating excellent separability of the extracted features. In Case A–E, both Linear SVM and RF achieved a perfect AUC of 100%, demonstrating complete discrimination between normal and seizure signals. In most cases, the performance of Linear SVM and RF is very close. RF slightly outperformed Linear SVM in Case B–E (99.99% vs. 99.76%) and marginally in Case D–E. However, Linear SVM achieved higher AUC values in Cases C–E, AB–E, and CD–E, with the most noticeable improvement observed in Case CD–E (99.80% vs. 99.29%). This suggests that Linear SVM provides better discrimination in more challenging combined-class scenarios. Although both classifiers demonstrate near-perfect classification performance, the Linear SVM shows slightly more consistent and robust AUC across complex cases, further supporting its selection as the optimal classifier for the proposed method.

**Table 5:** Comparison of AUC (%) performance between RF and Linear SVM on the Bonn EEG dataset.

Case	RF AUC	Linear SVM AUC
A vs. E	100.00	100.00
B vs. E	99.99	99.76
C vs. E	99.70	99.84
D vs. E	99.80	99.79
AB vs. E	99.62	99.82
CD vs. E	99.29	99.80

Linear SVM on the Bonn EEG dataset.

#### 5.1.4 Confusion matrix analysis

**Figure 5:**
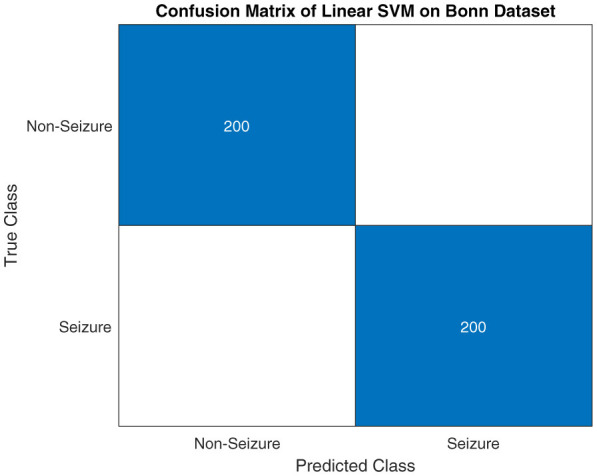
Confusion matrix of the linear SVM classifier for Case-1 (A vs. E) on the Bonn EEG dataset.

Figure 5 displays the confusion matrix of the Linear SVM for the Bonn EEG dataset. Seizure and non-seizure EEG segments are correctly classified in the confusion matrix. In particular, it correctly identified 200 non-seizure segments and 200 seizure segments, with no false positives or false negatives. In addition to confirming the strong discriminative capability of the suggested Hilbert-domain sub-band feature extraction framework in conjunction with mRMR feature selection and Linear SVM classification, this result shows 100% class separability in the classification instance under consideration.

### 5.2 Performance evaluation of CHB-MIT dataset

Table 6 and Table 7 presents the classification efficacy of the proposed seizure detection methodology on the CHB-MIT dataset employing five machine learning classifiers. The performance is assessed for each subject based on Accuracy (ACC), Sensitivity (SEN), and Specificity (SPEC). The table indicates that the proposed method consistently attains high accuracy across all subjects and classifiers. Among the classifiers, Linear SVM attained the greatest mean accuracy of 99.04%, followed by RF at 99.06%, ET at 99.04%, and LDA at 98.66%, while also maintaining superior precision and F1 scores. Furthermore, Linear SVM maintained a comparatively lower false-positive rate than the other classifiers, demonstrating its effectiveness in reducing false seizure detections in long-term EEG recordings. The RBF-SVM classifier also achieved excellent results and strong nonlinear classification ability. However, its performance was less consistent than Linear SVM for some subjects, especially in specificity and false positive rate. This also indicates that the proposed feature extraction and mRMR-based feature selection framework generates highly discriminative features that can be effectively separated by a simpler linear decision boundary. In terms of sensitivity, all classifiers demonstrate excellent performance, reflecting the model's ability to correctly identify seizure events. The ET classifier achieved the highest mean sensitivity of 99.76%, followed closely by RF (99.72%) and Linear SVM (99.71%), while LDA obtained 99.50%. RF and ET classifiers showed excellent sensitivity values that confirm the ability to correctly identify the seizure event. However, the FPR values were slightly higher for some subjects, which was attributed to class imbalance and subject-specific EEG variability. Some subjects, such as chb01, chb05, chb07, and chb09, obtained values close to 100% for precision and F1-score, indicating a good ability to discriminate seizures. Conversely, the difficulty of EEG recordings from subjects such as chb04 and chb06 led to relatively higher false-positive rates and lower specificity. This may be attributed to inter-subject EEG variability, imbalance between seizure and non-seizure samples, differences in seizure morphology, motion artifacts, and complex background EEG activity. These factors increase the overlap between seizure and non-seizure patterns, leading to comparatively higher false-positive predictions and lower specificity in some subjects. This indicates that the proposed feature-extraction and classification method is highly effective at detecting seizure segments, regarding specificity, which measures the ability to correctly identify non-seizure segments Linear SVM produced the best average specificity of 85.88%, outperforming ET (83.11%), RF (83.00%), and LDA (81.36%). Although the specificity values vary across subjects, Linear SVM consistently shows better discrimination between seizure and non-seizure signals. Subject-wise analysis shows that several subjects, such as Chb01, Chb05, Chb07, and Chb09, achieved extremely high accuracy values close to 100%, demonstrating the robustness of the proposed approach.

**Table 6:** Classification performance of the proposed method on the CHB-MIT dataset in terms of ACC (%), SEN, SPEC, PREC, F1, and FPR.

Subject	ET						RF						Linear SVM						LDA					
	ACC	SEN	SPEC	PREC	F1	FPR	ACC	SEN	SPEC	PREC	F1	FPR	ACC	SEN	SPEC	PREC	F1	FPR	ACC	SEN	SPEC	PREC	F1	FPR
Chb01	99.73	99.84	97.86	99.87	99.86	2.23	99.71	99.82	97.77	99.84	99.84	2.76	99.73	99.87	97.37	99.84	99.86	2.78	99.45	99.94	91.21	99.48	99.70	9.02
Chb02	99.35	99.91	73.23	99.38	99.64	27.03	99.32	99.89	72.60	99.36	99.63	28.17	99.86	99.99	93.50	99.84	99.92	6.77	99.58	99.79	88.87	99.79	99.78	9.63
Chb03	98.29	99.27	79.51	98.93	99.07	20.78	98.17	99.20	78.36	98.88	99.03	21.82	98.34	99.40	78.08	98.97	99.14	19.97	97.60	98.65	77.51	98.81	98.70	23.09
Chb04	98.20	99.81	66.13	98.36	99.07	33.53	98.14	99.71	66.80	98.39	99.04	32.98	98.74	99.75	78.47	98.91	99.32	22.16	97.96	99.14	74.44	98.76	98.92	25.24
Chb05	99.61	99.77	98.24	99.87	99.82	1.67	99.61	99.75	97.87	99.83	99.79	2.23	99.74	99.95	97.04	99.78	99.88	2.97	98.93	100.00	85.84	98.88	99.43	14.80
Chb06	99.30	99.92	58.00	99.28	99.59	50.00	99.29	99.90	58.86	99.19	99.54	54.67	98.79	99.62	43.43	98.83	99.29	78.00	98.16	98.76	58.50	99.37	99.03	42.00
Chb07	99.45	99.68	94.10	99.74	99.71	6.01	99.43	99.68	93.61	99.71	99.69	6.78	99.51	99.77	93.73	99.73	99.74	6.33	98.68	99.74	74.18	98.89	99.31	25.93
Chb08	97.43	99.52	82.05	97.65	98.56	17.89	97.98	99.38	82.82	97.78	98.56	16.87	97.69	99.05	87.69	98.31	98.67	12.70	97.56	99.24	85.22	98.07	98.64	14.61
Chb09	99.69	99.92	93.08	99.76	99.86	6.83	99.62	99.88	92.36	99.73	99.80	7.73	99.80	99.91	96.74	99.98	99.93	0.51	99.45	99.71	92.06	99.71	99.73	8.27
Chb10	99.38	100.00	88.96	99.37	99.68	10.96	99.36	99.98	88.95	99.39	99.68	10.62	99.46	99.86	92.72	99.59	99.75	6.99	99.20	100.00	85.79	99.18	99.58	14.18
**Average** **±** **STD**	**99.04** **±** **0.74**	**99.76** **±** **0.21**	**83.12** **±** **13.16**	**99.22** **±** **0.69**	**99.49** **±** **0.41**	**17.69** **±** **14.79**	**99.06** **±** **0.65**	**99.72** **±** **0.24**	**83.00** **±** **12.84**	**99.21** **±** **0.65**	**99.46** **±** **0.41**	**18.46** **±** **15.66**	**99.17** **±** **0.70**	**99.72** **±** **0.28**	**85.88** **±** **15.69**	**99.38** **±** **0.54**	**99.55** **±** **0.40**	**15.92** **±** **21.8**	**98.66** **±** **0.74**	**99.50** **±** **0.48**	**81.36** **±** **9.79**	**98.66** **±** **0.49**	**99.28** **±** **0.41**	**18.68** **±** **9.97**

Bold values indicate the best performance.

**Table 7:** RBF-SVM performance metrics on the CHB-MIT EEG dataset.

Subject	PREC (%)	F1 (%)	FPR (%)
Chb01	99.76	99.84	4.04
Chb02	99.73	99.86	11.77
Chb03	98.76	98.96	24.19
Chb04	98.86	99.30	23.20
Chb05	99.81	99.89	2.54
Chb06	99.40	99.57	42.00
Chb07	99.69	99.72	7.30
Chb08	98.34	98.78	12.53
Chb09	99.88	99.91	3.38
Chb10	99.58	99.76	7.24

The number of seizure segments and non-seizure EEG segments in the CHB-MIT dataset is significantly out of balance. Although explicit imbalance-handling techniques such as resampling or class weighting were not employed, a set of imbalance-aware evaluation metrics, including sensitivity, specificity, precision, F1-score, and false positive rate, was used instead of relying solely on accuracy. Additionally, the classifier robustness and discriminative power in unbalanced situations were enhanced by repeated k-fold cross-validation and mRMR-based feature selection. To improve the subject-independent seizure detection performance, class-weighted learning and sophisticated resampling strategies will be investigated in a future study.

#### 5.2.1 Statistical significance analysis

Friedman ANOVA indicated significant differences among classifiers for almost all subjects on the CHB-MIT EEG dataset, with p-values close to zero in most cases. The Tukey-Kramer post hoc analysis revealed that Linear SVM and RBF-SVM were consistently significantly better than LDA for most subjects shown in Table 8. Statistically significant improvements were also observed for SVM-based classifiers compared with tree-based methods in several subjects, including chb02, chb04, chb07, and chb09. However, for some subjects, such as chb05 and chb10, the performance differences between Linear SVM and RBF-SVM were not statistically significant, indicating that both classifiers performed equally well.

**Table 8:** Friedman ANOVA and Tukey-Kramer post hoc statistical analysis for different classifiers on the CHB-MIT EEG dataset.

Subject	Friedman *p*-value	Significant difference	Best classifier	Comparable classifiers
chb01	< 0.001	Yes	Linear SVM	ET, RF
chb02	< 0.001	Yes	Linear SVM	RBF-SVM
chb03	< 0.001	Yes	Linear SVM	ET
chb04	< 0.001	Yes	Linear SVM / RBF-SVM	–
chb05	< 0.001	Yes	RBF-SVM	Linear SVM
chb06	< 0.006	Yes	ET/RBF-SVM	RF
chb07	< 0.001	Yes	Linear SVM	RBF-SVM, ET
chb08	< 0.001	Yes	RBF-SVM	Linear SVM
chb09	< 0.001	Yes	Linear SVM	RBF-SVM
chb10	< 0.001	Yes	RBF-SVM	Linear SVM

Classifiers listed under Comparable Classifiers did not show statistically significant performance differences according to the Tukey–Kramer post-hoc analysis (*p*>0.05).

#### 5.2.2 ROC and AUC analysis

**Figure 6:**
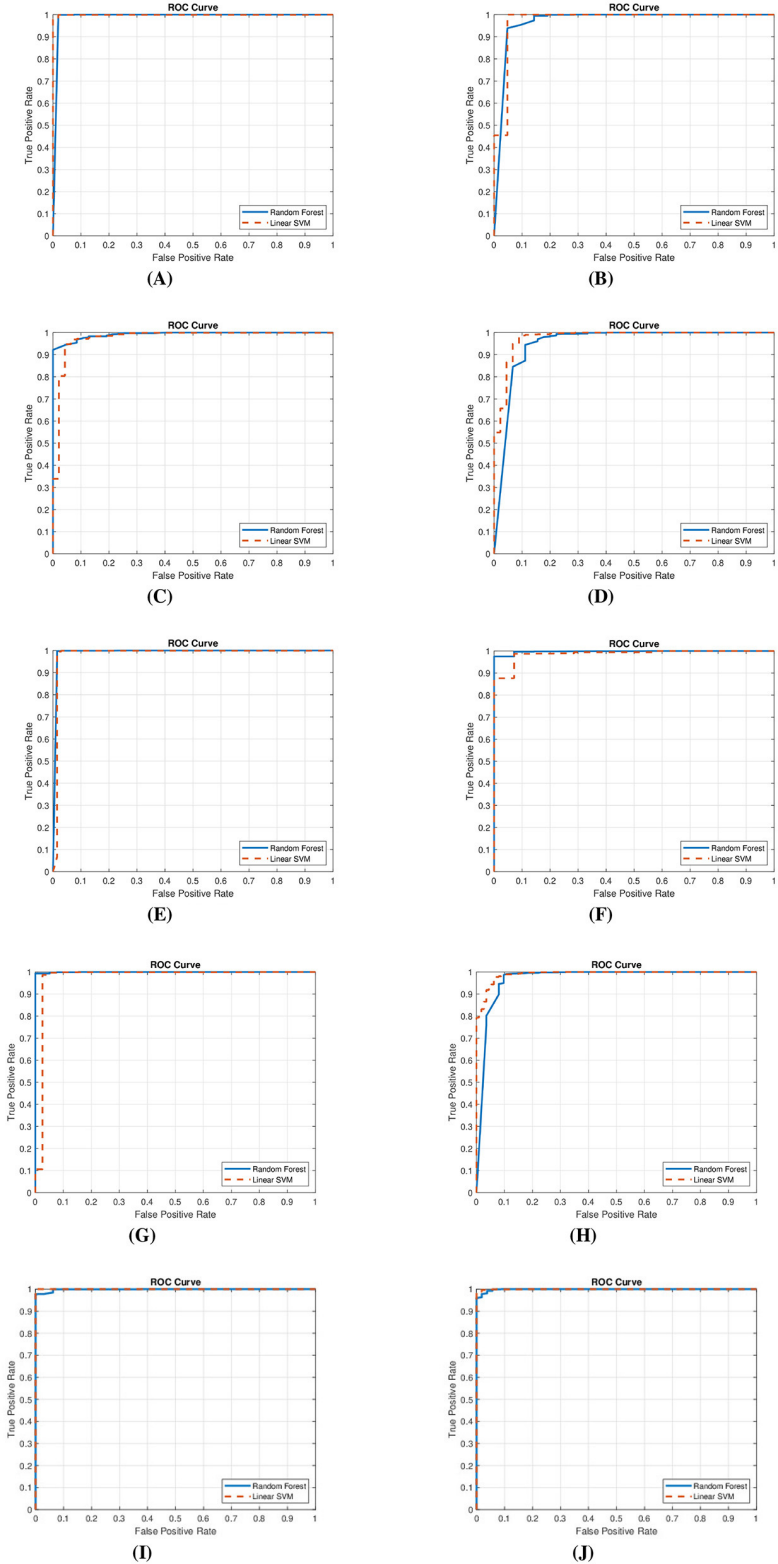
ROC curves for seizure detection on the CHB-MIT EEG dataset. **(A)** chb01. **(B)** chb02. **(C)** chb03. **(D)** chb04. **(E)** chb05. **(F)** chb06. **(G)** chb07. **(H)** chb08. **(I)** chb09. **(J)** chb10.

Figure 6 presents the ROC curves of the proposed seizure detection approach for different subjects in the CHB-MIT dataset. Both classifiers show curves very close to the upper-left corner shown in the figures, indicating excellent discrimination between seizure and non-seizure EEG segments. For most subjects (such as Chb01, Chb05, Chb07, Chb09, and Chb10), the ROC curves almost reach the ideal point [True Positive Rate (TPR) = 1] and [False Positive Rate (FPR) = 0], suggesting near-perfect classification capability. This analysis reveals that the proposed feature extraction and classification approach can effectively identify seizure events with minimal false alarms. A comparison between the classifiers shows that Random Forest and Linear SVM exhibit very similar ROC characteristics. However, in some subjects, the Linear SVM slightly outperforms Random Forest, achieving higher true positive rates at lower false positive rates. This demonstrates that both classifiers are highly effective for the proposed seizure detection framework. For a few subjects (e.g., Chb03, Chb04, and Chb08), the ROC curves show slightly larger FPR values due to the limited number of seizure events available in the dataset, which may lead to class imbalance and affect the classifier's ability to generalize effectively. Overall, the ROC analysis confirms that the proposed method achieves excellent classification performance across multiple subjects, with both Random Forest and Linear SVM providing high sensitivity and low false positive rates, thereby validating the effectiveness of the developed seizure detection system.

Table 9 presents the AUC values obtained using RF and Linear SVM classifiers for seizure detection on the CHB-MIT EEG dataset. From the table, both classifiers achieve very high AUC values across all subjects. Most subjects show AUC values greater than 95%, indicating reliable seizure detection performance. For several subjects, such as chb01, chb05, chb07, chb09, and chb10, both classifiers achieve AUC values close to 100%, which indicates near-perfect separation between seizure and non-seizure EEG signals. In particular, Random Forest achieves the highest AUC of 99.94% for subject chb07, while Linear SVM achieves the highest AUC of 99.89% for subject chb09.

**Table 9:** Comparison of AUC (%) performance between RF and Linear SVM on the CHB-MIT EEG dataset.

Subject	RF AUC	Linear SVM AUC
Chb01	99.80	99.76
Chb02	95.90	95.63
Chb03	98.60	97.70
Chb04	95.42	97.54
Chb05	99.25	99.69
Chb06	99.84	99.03
Chb07	99.94	99.67
Chb08	96.51	98.84
Chb09	99.58	99.89
Chb10	99.70	99.43

#### 5.2.3 Confusion matrix analysis

**Figure 7:**
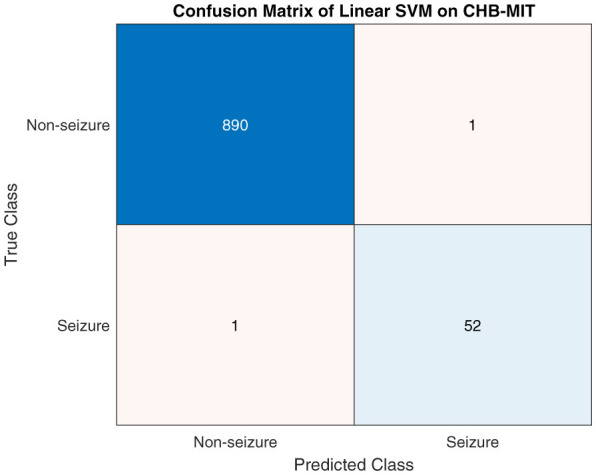
Confusion matrix of the linear SVM classifier for subject Chb01 on the CHB-MIT EEG dataset.

The confusion matrix produced by the Linear SVM classifier on the CHB-MIT EEG dataset is displayed in Figure 7. Seizure segments 52 and non-seizure segments 890 were accurately categorized by the proposed framework. There was just one instance of a non-seizure segment being incorrectly categorized as a seizure and one instance of a seizure section being incorrectly labeled as a non-seizure. The findings demonstrate that the proposed architecture can maintain strong seizure-detection performance while achieving extremely low false-positive and false-negative rates. The utility of derived Hilbert-domain sub-band features in differentiating seizure from non-seizure EEG patterns in long-term clinical EEG recordings is further validated by the confusion matrix.

#### 5.2.4 Subject-wise validation

To evaluate the generalization capability of the proposed framework on unseen patients, a subject-independent Leave-One-Subject-Out (LOSO) validation strategy was employed for the CHB-MIT dataset. In this protocol, EEG data from one subject were used exclusively for testing, while the remaining subjects were used for training. This procedure was repeated iteratively for all 10 subjects, ensuring that no EEG segments from the test subject appeared in the training set, and the corresponding testing performance for each unseen subject is reported. Table 10 summarizes the subject-independent LOSO validation performance of the proposed framework on the CHB-MIT EEG dataset. The results reveal considerable variation among subjects, primarily due to natural differences in EEG characteristics and seizure patterns. The proposed framework showed strong performance across several subjects, particularly chb01, chb02, chb05, chb07, and chb09, with classification accuracy exceeding 92%. Among these subjects, chb02 and chb04 achieved 100% sensitivity, meaning that all seizure events were successfully detected. Furthermore, chb05 and chb09 achieved 100% precision and specificity, indicating that no false-positive predictions were generated in these cases. On the other hand, comparatively lower performance was observed for subjects such as chb03, chb04, chb06, chb08, and chb10. In particular, chb06 achieved the lowest accuracy, 46.43%, along with lower sensitivity and specificity. This suggests that seizure and non-seizure EEG patterns for this subject were more difficult to distinguish. Similarly, chb04 exhibited a relatively low specificity of 35.56% and a high false-positive rate of 64.44%, indicating that many normal EEG segments were incorrectly identified as seizure activity. One of the major challenges associated with the CHB-MIT dataset is the significant class imbalance between seizure and non-seizure EEG segments. Since non-seizure samples greatly outnumber seizure samples, the classifier may become biased toward the majority class, reducing its ability to generalize effectively across subjects. Furthermore, variations in seizure duration, recording environments, electrode placement, and patient-specific EEG characteristics further complicate subject-independent seizure detection. Overall, the proposed framework achieved an average accuracy of 81.73%, sensitivity of 84.22%, specificity of 77.34%, precision of 81.75%, and F1-score of 81.45%. The average false positive rate was 22.66%. These results demonstrate that the proposed method performs effectively for most subjects, although inter-subject EEG variability and dataset imbalance continue to pose significant challenges for generalized seizure detection.

**Table 10:** Cross-subject performance of the proposed model using the CHB-MIT dataset.

Test subject	ACC (%)	SEN (%)	SPEC (%)	PREC (%)	F1 (%)	FPR (%)
Chb01	98.11	98.11	98.11	98.11	98.11	1.89
Chb02	97.62	100.00	95.24	95.46	97.67	4.76
Chb03	75.53	97.87	53.19	67.65	80.00	46.81
Chb04	67.78	100.00	35.56	60.81	75.63	64.44
Chb05	93.38	86.77	100.00	100.00	92.91	0.00
Chb06	46.43	42.86	50.00	46.15	44.44	50.00
Chb07	94.87	97.44	92.31	92.68	95.00	7.69
Chb08	69.91	83.19	56.64	65.73	73.44	43.36
Chb09	92.19	84.38	100.00	100.00	91.53	0.00
Chb10	75.47	56.60	94.34	90.91	69.77	5.66
**Average** **±** **STD**	**81.73** **±** **16.1**	**84.22** **±** **18.79**	**77.34** **±** **24.08**	**81.75** **±** **18.67**	**81.45** **±** **16.01**	**22.66** **±** **24.01**

Bold values indicate the best performance.

### 5.3 Computational complexity and runtime analysis

Sub-band decomposition, Hilbert Transform-based feature extraction, mRMR feature selection, and classifier training/testing are the primary contributors to the computational difficulty of the proposed framework. The proposed method is based on the extraction of handcrafted features and conventional machine learning classifiers; its computational cost is significantly lower than that of deep learning-based methods, which require millions of trainable parameters and GPU-intensive optimization. The execution time of each classifier was monitored across multiple experimental runs on the Bonn and CHB-MIT datasets to evaluate runtime performance. The findings demonstrate that Linear SVM offers the optimal balance between classification performance and computation. For all classification cases in the Bonn EEG dataset, the average execution time of the Linear SVM was between 0.22 and 1.54 seconds, while the RBF-SVM took significantly longer, between 23 and 61 seconds, due to nonlinear kernel computation and hyperparameter optimization. Similarly, with the CHB-MIT dataset, RBF-SVM required 10 to 62 seconds per subject, depending on subject complexity, whereas Linear SVM required only 0.05 to 0.32 seconds per subject. These findings demonstrate that Linear SVM is far more appropriate for applications requiring near real-time seizure monitoring and low latency. Since only a few handcrafted features are extracted from each EEG segment prior to dimensionality reduction using mRMR feature selection, the proposed framework remains computationally light in terms of memory requirements. This significantly reduces memory usage and feature dimensionality for classification. The proposed approach can be implemented on ordinary clinical workstations and resource-constrained edge devices because it does not require large-scale model parameter storage or high-capacity GPU memory like deep learning frameworks.

### 5.4 Ablation study

An ablation study was performed to evaluate the individual contribution of each major component of the proposed framework. Four methodological configurations were investigated: Hilbert Transform only, Sub-band Decomposition only, Hilbert Transform with Sub-band Decomposition, and the complete proposed framework integrating Hilbert Transform, Sub-band Decomposition, and Feature Selection. As shown in Table 11, the Hilbert Transform-only approach achieved an average accuracy of 96.84% on the Bonn EEG dataset. When sub-band decomposition was used independently, the average accuracy increased to 98.80%, indicating that frequency-specific decomposition provides more discriminative information for seizure classification. The combination of Hilbert Transform and Sub-band Decomposition further improved the average accuracy to 98.82%. The highest average accuracy, 99.27%, was obtained when feature selection was incorporated into the Hilbert- and sub-band-based features. This result demonstrates that feature selection removes redundant and less informative features, thereby improving classification performance. A similar trend was observed for the CHB-MIT dataset, as presented in Table 12. The Hilbert Transform-only method achieved an average accuracy of 97.94%, while Sub-band Decomposition alone improved the performance to 98.59%. Combining Hilbert Transform and Sub-band Decomposition further increased the accuracy to 98.68%. The complete proposed framework achieved the best average accuracy of 99.04%, confirming the effectiveness of integrating time-frequency feature extraction with feature selection. Furthermore, Friedman's statistical tests were conducted to examine whether the performance differences among the four configurations were statistically significant. The obtained *p*-values were 0.002 for the Bonn dataset and 0.004 for the CHB-MIT dataset, both of which are less than 0.05. Therefore, the differences among the compared methods were statistically significant. These findings confirm that each component contributes positively to overall performance and that the complete framework provides the most robust and accurate seizure detection across both datasets.

**Table 11:** Accuracy comparison of different methods on the Bonn EEG dataset.

Case	Hilbert transform only (%)	Sub-band decomposition only (%)	Hilbert + sub-band decomposition (%)	Hilbert + dub-band + feature selection (%)
A vs. E	98.23	99.99	99.81	100.00
B vs. E	96.04	99.14	99.26	99.75
C vs. E	97.22	98.10	99.18	98.87
D vs. E	96.49	98.40	98.35	98.61
AB vs. E	97.52	98.40	98.20	99.70
CD vs. E	95.54	98.78	98.12	98.67
**Average** **±** **STD**	**96.84** **±** **0.91**	**98.80** **±** **0.62**	**98.82** **±** **0.63**	**99.27** **±** **0.51**

Bold values indicate the best performance.

**Table 12:** Accuracy comparison of different methods on the CHB-MIT EEG dataset.

Subject	Hilbert transform only (%)	Sub-band decomposition only (%)	Hilbert + sub-band decomposition (%)	Hilbert + sub-band + feature selection (%)
Chb01	99.57	99.41	99.18	99.73
Chb02	97.88	99.58	99.29	99.35
Chb03	97.44	98.45	98.14	98.29
Chb04	96.85	98.78	97.08	98.20
Chb05	97.67	97.56	98.17	99.61
Chb06	98.52	98.20	98.45	99.30
Chb07	98.60	99.23	99.45	99.45
Chb08	96.94	97.13	97.59	97.43
Chb09	98.23	99.29	99.89	99.69
Chb10	97.77	98.23	99.52	99.38
**Average** **±** **STD**	**97.94** **±** **0.78**	**98.59** **±** **0.78**	**98.68** **±** **0.88**	**99.04** **±** **0.70**

Bold values indicate the best performance.

An ablation study was also performed to evaluate the effectiveness of spike-related features used in the proposed framework. The Fluctuation Index (FI), Coefficient of Variation (CV), and SODP-based ellipse area characteristics make up the spike-related feature group. Tables 13, 14 demonstrate the high discriminative potential of these features for seizure detection on the CHB-MIT EEG dataset as well as the Bonn dataset. The competitive classification performance of spike-related characteristics demonstrated their efficacy in characterizing abrupt amplitude changes and transitory spike patterns associated with epileptic episodes. Additionally, the coupling of entropy-based and statistical features with spike-related features enhances the overall classification performance, confirming that the spike-related features are crucial to the robustness of the proposed seizure detection system. The irregularity and complexity of EEG signals, which usually increase during epileptic seizure activity, are quantified by entropy-based features. Seizure and non-seizure EEG patterns can be distinguished using statistical criteria that capture amplitude distribution, variance, asymmetry, and signal power characteristics. Furthermore, spike-related features are especially useful for detecting sudden waveform changes and brief spike activity frequently seen during seizures. The proposed framework improves overall seizure classification performance by capturing both nonlinear dynamics and morphological EEG characteristics thanks to the complementary nature of these three feature categories.

**Table 13:** Performance analysis of spike-related features with linear SVM for different cases on the Bonn EEG dataset.

Case	ACC (%)	SEN (%)	SPEC (%)	PREC (%)	F1-score (%)
A vs. E	99.50	99.00	100.00	100.00	99.48
B vs. E	99.00	98.00	100.00	100.00	98.95
C vs. E	99.23	98.47	100.00	100.00	99.20
D vs. E	97.93	96.40	99.47	99.49	97.84
AB vs. E	99.34	98.03	100.00	100.00	98.97
CD vs. E	98.38	95.63	99.75	99.52	97.45
**Average** **±** **STD**	**98.98** **±** **0.56**	**97.58** **±** **1.18**	**99.87** **±** **0.20**	**99.84** **±** **0.23**	**98.65** **±** **0.74**

Bold values indicate the best performance.

**Table 14:** Performance analysis of spike-related features with linear SVM for different cases on the CHB-MIT EEG dataset.

Case	ACC (%)	SEN (%)	SPEC (%)	PREC (%)	F1-score (%)
Chb01	99.43	99.76	93.91	99.64	99.70
Chb02	99.77	100.00	89.30	99.76	99.88
Chb03	98.41	99.44	78.68	98.90	99.16
Chb04	96.28	99.38	34.42	96.82	98.08
Chb05	99.28	99.79	92.71	99.45	99.61
Chb06	98.52	100.00	0.00	98.52	99.25
Chb07	99.30	99.76	88.61	99.52	99.64
Chb08	97.10	99.36	80.45	97.43	98.37
Chb09	99.76	99.87	96.57	99.88	99.87
Chb10	99.56	99.97	92.64	99.57	99.77
**Average** **±** **STD**	**98.74** **±** **1.13**	**99.73** **±** **0.24**	**74.73** **±** **30.25**	**98.95** **±** **1.0**	**99.33** **±** **0.60**

Bold values indicate the best performance.

## 6 Discussion

In this study, nonlinear features and entropy-based features are combined, and the mRMR feature selection method is employed with a machine learning classifier to detect the seizure events in EEG signals. The simulation results demonstrate that the proposed method performs well in classifying epileptic seizures. The results demonstrate that the Linear SVM model achieved superior overall performance across all classification scenarios. Specifically, it attained an average accuracy of 99.27% on the Bonn dataset and 99.04% using the CHB-MIT dataset. These observations confirm that handcrafted feature-based approaches remain highly effective on the Bonn EEG dataset, which is relatively structured and less noisy than real-world multi-channel clinical EEG recordings. Among the deep learning models, CNN and Transformer showed competitive performance, particularly in the A vs. E and AB vs. E cases. The strong performance of CNN can be attributed to its ability to effectively capture local temporal patterns within EEG signals. Similarly, the Transformer demonstrated robust performance in several cases, likely due to its capability to model long-range temporal dependencies through self-attention mechanisms. Conversely, the LSTM model achieved significantly lower accuracy across most cases. This may be due to the difficulty of learning long temporal dependencies from relatively short EEG segments or the limited size of the Bonn dataset, which may not provide sufficient training data for recurrent architectures to generalize effectively. The grouped cases (AB vs. E and CD vs. E) generally yielded improved performance compared to individual class comparisons, suggesting that combining non-seizure classes increases separability from seizure signals. Overall, the findings indicate that while deep learning models provide competitive performance, traditional machine learning methods, such as Linear SVM, can still outperform complex neural architectures on structured EEG datasets with limited sample sizes. The findings presented in Tables 2, 6 indicate that all classifiers attained superior classification performance, validating the efficacy of the extracted EEG features. Among the evaluated classifiers, Linear SVM demonstrated the most consistent and overall best performance in terms of accuracy, sensitivity, specificity, and AUC. Its advantage was more evident in the more challenging combined cases (AB vs. E and CD vs. E), where reliable discrimination is critical. Although RF achieved very similar performance and even slightly higher AUC in a few individual cases, Linear SVM showed better overall stability across all scenarios. One possible reason is that the proposed Hilbert-domain sub-band features combined with mRMR feature selection produced a highly discriminative feature space with improved class separability. As a result, a linear decision boundary became sufficient for effective classification. Furthermore, Linear SVM generally provides better generalization performance and a lower risk of overfitting in high-dimensional EEG feature spaces compared to nonlinear kernels. The reduced computational complexity and improved stability of Linear SVM also contributed to its superior overall performance across different seizure detection scenarios. Additionally, the consistently high specificity values obtained by all classifiers confirm their strong ability to correctly identify non-seizure signals. Based on these observations, the Linear SVM is considered the most suitable model for seizure detection in this study. The superior classification performance can be attributed to the Hilbert-domain representation over narrowband EEG signals. By extracting instantaneous amplitude and phase-related features from sub-band components, the proposed method captures transient seizure characteristics more effectively than traditional time- or frequency-domain approaches.

The classification performance of the proposed seizure EEG signal detection method is compared to other recently developed methods in this section. Table 15 summarizes the comparison with the proposed method, using the Bonn University dataset. The average accuracy across all scenarios must be considered when comparing a method's overall performance. Table 15 shows that the proposed method's average accuracy across all cases is higher than that of the previously developed algorithms. The highest classification accuracy, 100%, was achieved for case A vs. E, B vs. E, and AB vs. E in Mardini et al. (2020), while the average classification accuracy of our proposed method is 1.13% higher than that reported in Mardini et al. (2020). In the CD vs. E case, the proposed method achieved 3.83% higher performance than that reported in Mardini et al. (2020). The accuracy for C vs. E and D vs. E of all other methods in Table 15 is lower than that of our proposed method. Six different cases are considered in Zhao et al. (2020), Sriraam et al. (2018), and Mardini et al. (2020) and achieve average classification accuracies of 98.61%, 97.44%, and 97.95%, which are 0.47%, 1.64%, and 1.13% lower than those of our proposed method. Sadiq et al. (2024) mentioned only a mean accuracy of 96.88%, which is 2.39 lower than our proposed method. In terms of overall performance, with the lowest standard deviation, the proposed approach outperforms all recently established algorithms. Table 16 presents a comparison between the proposed method and several recently published seizure detection approaches evaluated on the CHB-MIT dataset. The results indicate that the proposed framework achieves 99.04% accuracy, outperforming several recent deep learning and transformer-based models reported in the literature. This improvement demonstrates the effectiveness of the proposed feature extraction and mRMR feature selection combined with the Linear SVM classifier for reliable seizure detection.

**Table 15:** Comparison of the performance of the proposed method with recently developed algorithms on the Bonn EEG dataset in terms of accuracy (%).

References	Methods	Classifier	Cases	Accuracy (%)
Gupta and Pachori (2019)	Entropy of FBSE	LS-SVM	A–E	99.50
			B–E	99.50
			D–E	97.50
			Mean ± STD	98.83 ± 0.94
Sriraam et al. (2018)	Matrix-determinant-based features	MLPNN	A–E	99.45
			B–E	96.06
			C–E	97.60
			D–E	97.60
			AB–E	97.10
			CD–E	96.85
			Mean ± STD	97.44 ± 1.04
Zhao et al. (2020)	Deep neural network	CNN	A–E	99.52
			B–E	99.11
			C–E	98.02
			D–E	97.63
			AB–E	99.38
			CD–E	98.03
			Mean ± STD	98.61 ± 0.74
Mardini et al. (2020)	DWT + genetic algorithm	ANN	A–E	100.00
			B–E	100.00
			C–E	95.00
			D–E	98.30
			AB–E	100.00
			CD–E	94.40
			Mean ± STD	97.95 ± 2.38
Qureshi et al. (2022)	Time domain + spectrum + nonlinear features + LBP	Feedforward NN	A–E	96.67
			B–E	91.67
			C–E	91.67
			D–E	85.00
			AB–E	90.00
			CD–E	91.11
			Mean ± STD	91.02 ± 3.42
Liu et al. (2023)	Time-frequency + DBM (unsupervised)	SVM	A–E	100.00
			B–E	98.60
			C–E	96.70
			D–E	94.20
			AB–E	95.90
			CD–E	95.90
			Mean ± STD	96.88 ± 1.91
Sadiq et al. (2024)	Hellinger distance + PSO	Hellinger	Mean	96.88
Alzamili et al. (2025)	Time domain + Ruzicka FS	RF	A–E	97.50
			B–E	100.00
			C–E	100.00
			D–E	100.00
			AB–E	90.00
			CD–E	81.86
			Mean ± STD	94.86 ± 6.88
**Proposed method**	**Hilbert transform + nonlinear + entropy + mRMR**	**Linear SVM**	A–E	99.97
			B–E	99.51
			C–E	98.73
			D–E	98.37
			AB–E	99.63
			CD–E	98.24
			**Mean** **±** **STD**	**99.08** **±** **0.66**

Bold values indicate the best performance.

**Table 16:** Performance comparison of the proposed method with recent seizure detection methods on the CHB-MIT dataset in terms of accuracy (%).

References	Methods	Classifier	Accuracy (%)
Rukhsar and Tiwari (2023)	Lightweight convolution transformer	Transformer	96.31
Shen et al. (2024)	STFT GoogleNet	CNN	97.74
Lu et al. (2025)	Mamba-based SlimSeiz network	Deep learning	94.80
Gao et al. (2025)	Time-frequency dual stream transformer with gated attention	Transformer	95.18
**Proposed method**	**Hilbert transform + nonlinear + entropy-based features with mRMR feature selection**	**Linear SVM**	**99.04**

Bold values indicate the best performance.

**Figure 8:**
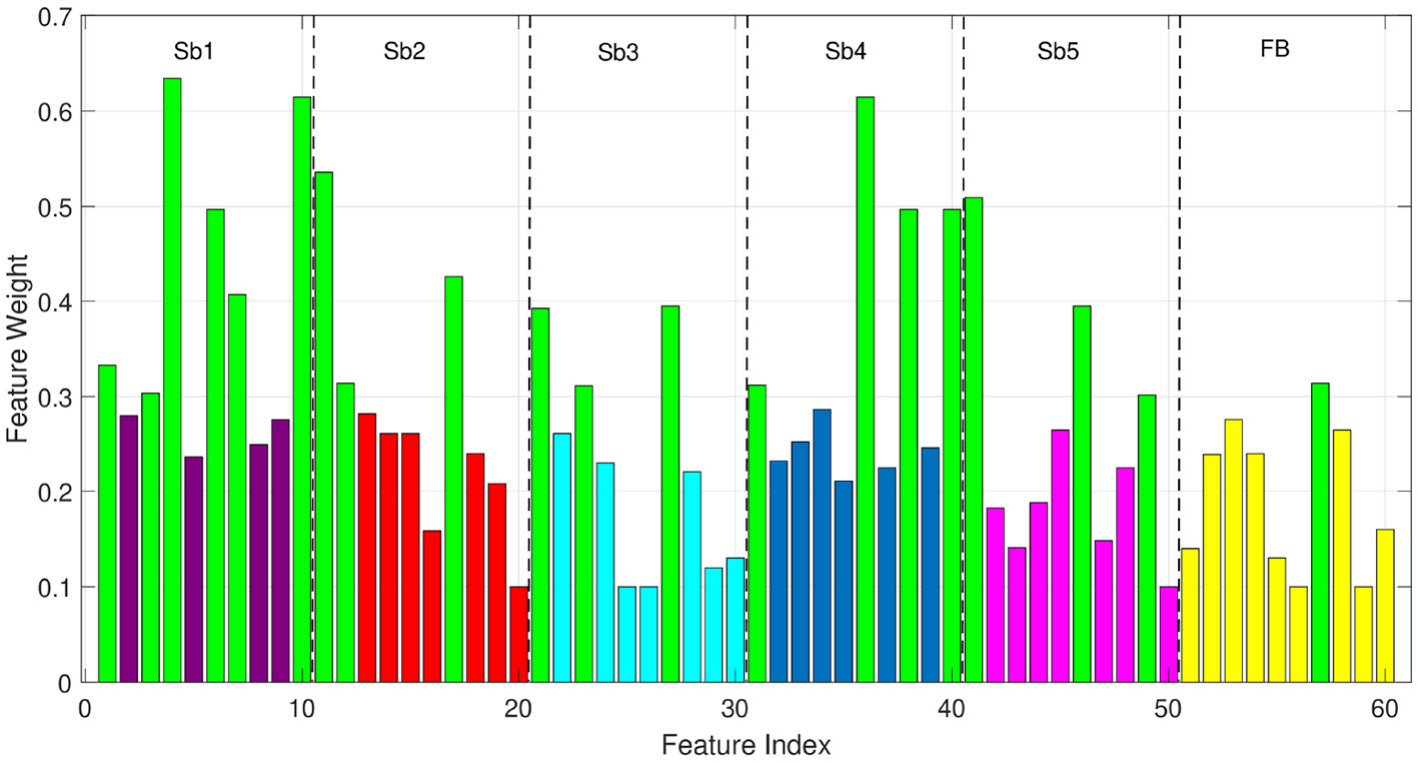
The features from different sub-bands (indicated by different colors) (*X* axis) with corresponding feature weights (*Y* axis). The 20 features are selected (marked in green) from different sub-bands based on their weights. *Sb*1−*Sb*5 represent five sub-bands, and *FB* denotes the full-band EEG from the Bonn dataset. Each sub-band consists of ten features, ordered as follows: Shannon entropy, coefficient of variation (CV), fluctuation index (FI), second-order difference plot (SODP), Renyi's entropy, Tsallis entropy, log energy entropy, Hjorth parameters, kurtosis, and skewness.

The number of selected features significantly impacts the efficacy of the proposed method. In this proposed method, three categories of features are combined, and a subset of features is selected using the mRMR feature selection algorithm. The features are ranked based on how well they discriminate between classes. Using the mRMR feature selection method, each feature is assigned a weight. To maximize performance, several high-ranking features are selected for seizure detection. To achieve the maximum accuracy, a grid search method is used to select the number of features. Figure 8 demonstrates the weights assigned by mRMR to each of the 60 features of the case (C-E). The weight assigned to each feature vector is used to sort the feature vectors. The top 20 ranked features (green color) are selected (from the sorted feature vector) for seizure detection. The remaining features are irrelevant or redundant and have been removed to reduce performance degradation. It also reduces machine learning training. Based on the mRMR feature ranking results, the final number of selected features is fixed at 20 for each dataset, as this configuration yields the highest classification accuracy. The effectiveness of sub-band features is utilized here. Features were selected using mRMR across sub-bands as follows: six from Sb1, three from Sb2, three from Sb3, four from Sb4, and three from Sb5, supplemented by one feature from the full-band (FB) signal as shown in Figure 8 for case C vs. D from the Bonn dataset. Based on the feature selection results, entropy-based features emerged as the most dominant and effective feature category for characterizing the EEG dataset, accounting for 12 out of the 20 selected features. It is observed that the full-band EEG signal is less effective at detecting seizure events when only one feature is selected from it. This evidence shows that the sub-band features effectively improve the performance of the proposed method. The sub-band features are discriminative components that are found in narrowband signals. Although the proposed framework achieved high classification performance, it is important to acknowledge several limitations of the current study. First, the Bonn EEG dataset is a relatively controlled benchmark dataset that may yield higher classification accuracies than more complex and heterogeneous real-world clinical EEG recordings. Therefore, the reported performance may not fully reflect practical clinical conditions, in which EEG signals often exhibit significant inter-subject variability, noise, and recording artifacts. Furthermore, the evaluation was primarily conducted using publicly available benchmark datasets with limited subject diversity. The proposed framework was also assessed in an offline experimental setting rather than in continuous real-time EEG monitoring environments. As a result, issues related to latency, computational efficiency, and long-term real-time deployment were not fully investigated. Furthermore, although the handcrafted feature extraction strategy demonstrated strong discriminative capability, additional optimization may be necessary for implementation in wearable or embedded healthcare devices with limited computational resources. To further enhance the clinical applicability and robustness of the proposed framework, future research will focus on subject-independent seizure detection, online real-time EEG processing, and validation using larger multi-center clinical EEG datasets collected under more realistic conditions.

## 7 Conclusion

Due to subject dependence and a lack of available training data, automatic detection of epileptic seizures in EEG is a difficult task. Due to the non-linear and non-stationary nature of EEG signals, this study decomposed them into narrow sub-bands to extract features. To assess the efficiency and effectiveness of the proposed method, publicly available EEG datasets are used. The main contribution of this study is the use of the Hilbert Transform on a narrowband signal for feature extraction and mRMR for feature selection to detect the epileptic seizure. The performance of the proposed method is compared to the state-of-the-art algorithms. The results indicate that the proposed framework achieves competitive performance compared to existing approaches for seizure-vs.-non-seizure classification. Furthermore, the proposed method provides a more efficient and easily interpretable alternative to deep learning models. Using a smaller set of meaningful handcrafted features, along with classical machine learning techniques, reduces computational complexity while maintaining clarity. This makes it especially well-suited for real-world applications, particularly in environments where resources are limited. Future work will focus on validating the proposed framework using larger and more clinically diverse EEG datasets to further assess real-world generalization capability. Additionally, hyperparameter optimization for RF and ET classifiers could further improve performance. Future work may also investigate advanced optimization strategies and deep kernel learning approaches.

## Data Availability

The original contributions presented in the study are included in the article/supplementary material, further inquiries can be directed to the corresponding authors.
